# War-related urological trauma in Israel: a retrospective national registry analysis

**DOI:** 10.1007/s00345-025-05899-5

**Published:** 2025-09-02

**Authors:** Husny Mahmud, Yoram Mor, Adi Givon, Noam Kitrey, Israel Trauma Group (ITG), H. Bahouth, M. Bala, A. Bar, A. Braslavsky, D. Czeiger, D. Fadeev, A. L. Goldstein, I. Grevtsev, E. Hashavia, G. Hirschhorn, I. Jeroukhimov, A. Kedar, Y. Klein, A. Korin, B. Levit, U. Neeman, I. Schrier, A. D. Schwarz, W. Shomar, O. Yaslowitz, I. Zoarets

**Affiliations:** 1 Department of Urology, The Chaim Sheba Medical Center, Hashomer, 52621 Ramat Gan, Israel; 2 Gray Faculty of Medical and Health Sciences, Tel Aviv University, Tel Aviv, Israel; 3 Gertner Institute of Epidemiology and Health Policy Research, Sheba Medical Center, Tel-Hashomer, Ramat Gan, Israel

**Keywords:** Urological trauma, War injuries, Explosive injuries, Gunshot wounds, Rehabilitation, Trauma surgery

## Abstract

**Background:**

Urological trauma significantly affects patient morbidity, functional outcomes, and healthcare resource utilization. This study evaluated war-related genitourinary (GU) injuries that occurred during the Israeli–Gaza War, with a focus on injury patterns, management, and clinical outcomes.

**Methods:**

We conducted a retrospective analysis utilizing the Israeli National Trauma Registry, which is managed by the Gertner Institute for Health Policy and Epidemiology. This registry captures data on all of the patients admitted to trauma centers in Israel. Data quality is maintained through ongoing training of data abstractors and regular audits of registry data. All documented hospitalizations from October 7, 2023, to May 31, 2024, were included, excluding noncombat-related injuries. GU injuries were classified by anatomical location into the upper urinary tract (kidney/ureter), lower urinary tract (bladder/urethra), and external genitalia (penis/scrotum/testes). The data collected included demographics, injury mechanisms, injury severity score (ISS), surgical management, and clinical outcomes (ICU admissions, hospital length of stay [LOS], rehabilitation needs, and mortality). GU patients were compared with non-GU patients.

**Results:**

Of the 2,422 total casualties, 117 (4.8%) sustained GU injuries. The mean age was *27.2 ± 9.2 years* (range 14–76) in the GU-injured casualty group versus *30.1 ± 15.1 years* (range 0–97) in the non-GU casualty group; the difference was not significant (*p* = 0.43), with 97.4% males. Explosive devices (59.0%) and gunshot wounds (36.8%) were the predominant injury mechanisms. Severe injuries (ISS ≥ 16) occurred significantly more frequently among GU-injured patients (61.5% vs. 21.6%, p < 0.0001). External genital injuries were the most common injuries (60.7%), followed by kidney (27.4%) and bladder damage (10.3%). Associated pelvic fractures occurred in 18% of the GU trauma patients versus 1.8% of the non-GU-injured patients (p < 0.0001). Surgical interventions were performed in 84.6% of the patients, among whom scrotal/testicular surgeries (27.4%) were most common. ICU admissions (44.4%), prolonged hospitalizations ≥ 14 days (41.4%), rehabilitation needs (53.0%), and mortality (6.8%) were significantly greater among GU-injured patients.

**Conclusions:**

War-related GU trauma, although relatively uncommon, involves severe injuries, extensive surgical management, prolonged hospitalizations, and substantial rehabilitation demands. Enhancements in protective equipment and multidisciplinary treatment strategies are critical for optimizing outcomes in future conflicts.

## Background

War-related genitourinary (GU) injuries, although comprising a minority of overall combat casualties, represent a unique subset of trauma with the potential for significant morbidity and long-term disability [1–3]. Historically, their reported incidence has ranged from approximately 1% to 5% of all war injuries, varying by the nature of the conflict, available medical support, and protective measures [1, 4]. Despite their relatively small proportion, urological trauma can lead to extensive damage to the kidneys, ureters, bladder, urethra, and external genitalia (penis and scrotum), potentially compromising urinary and sexual function, reproductive potential, and overall psychological well-being [5–7].

Modern combat environments increasingly feature various devices with high-energy mechanisms, such as explosive devices and high-velocity firearms [1, 4]. These mechanisms produce multisystem injuries that often involve multiple organ systems, including the GU tract [2, 3]. The extent of damage is frequently exacerbated by evolving wartime tactics—ranging from improvised explosive devices (IEDs) and large-scale rocket fires to close-quarters engagements—which heighten the risk of penetrating and blast injuries [8].

The Israeli–Gaza War, initiated on October 7, 2023, exemplifies this trend. The conflict was characterized by the widespread use of explosive devices and rocket fires in densely populated civilian areas, posing unique challenges for medical management and contributing to the pattern of GU injuries observed [9]. These challenges included difficulties in the timely evacuation of casualties, potential resource limitations in overwhelmed medical facilities, and the complexity of injuries resulting from high-energy explosive mechanisms.

While improvements in body armour have reduced thoracic and abdominal injuries in many conflicts, they often leave the pelvic region less protected, resulting in a relative increase in injuries to the external genitalia and lower urinary tract [3, 10, 11]. The Israeli–Gaza War exemplifies this trend, with a significant number of casualties sustaining GU injuries despite the use of body armor [9].

Recent multinational military analyses have reinforced these patterns. Data from the International Security Assistance Force (ISAF) mission in Afghanistan demonstrated a predominance of external genital injuries, often accompanied by pelvic and extremity trauma [12]. Similarly, a contemporary analysis of 25,897 casualties from the U.S. Department of Defense Trauma Registry (DoDTR) (2007–2020) reported a 7.2% incidence of GU injuries, most commonly scrotal and testicular, with high survival rates but substantial rehabilitation needs [13].

Although case reports and smaller data analyses have documented GU trauma in recent conflicts, ongoing large-scale, systematically collected data remain crucial to optimize clinical protocols, shape triage guidelines, and facilitate enhancements in protective equipment [4, 7]. By synthesizing these data, we aim to provide stakeholders—clinicians, policymakers, and military personnel—with concrete, evidence-based insights to improve the prevention and management of war-related GU trauma.

## Methods

### Study design and population

We conducted a retrospective review using data from the Israeli National Trauma Registry (INTR), which prospectively collects data on all patients admitted to trauma centers in Israel, including both civilian and military facilities. The registry maintains data quality through ongoing training of abstractors and regular audits. Our study covered the period October 7, 2023, to May 31, 2024, and included all patients with documented GU injuries—encompassing the upper tract (kidney, ureter), lower tract (bladder, urethra), or external genitalia (penis, scrotum). Objectives were to (1) determine incidence and anatomical distribution, (2) evaluate severity and mechanisms of injury, (3) assess operative versus conservative management, and (4) analyze short-term outcomes including ICU admission, length of stay, rehabilitation needs, and mortality.

### Data collection

The collected variables included demographic characteristics (age, sex), mechanisms of injury (explosive devices, gunshot wounds, and other mechanisms), injury severity scores (ISSs, categorized as < 16 or ≥ 16), anatomical locations of GU injuries (kidney, ureter, bladder, urethra, penis, scrotum/testes), surgical management procedures (nephrectomy, orchiectomy, organ‑sparing procedures, among others), and clinical outcomes (ICU admission, hospital LOS, discharge outcomes—home, rehabilitation, or death—and overall mortality).

### Statistical analysis

Descriptive statistics were employed to characterize the study population. The means (± SDs) or medians (IQRs) were used for continuous variables, and categorical data were summarized as frequencies and percentages. Group comparisons (e.g., ISS ≥ 16 vs. ISS < 16) were performed with chi-square or Fisher’s exact tests for categorical variables and t tests or rank-sum tests for continuous variables, as appropriate. Significance was established at p < 0.05.

We performed a multivariable logistic regression with ICU admission (yes/no) as the dependent variable. The candidate predictors were age (per decade), injury severity score ≥ 16, injury mechanism (explosive vs other) and the presence of any genitourinary (GU) injury.

### Ethical considerations

This study was approved by the Institutional Review Board/Ethics Committee (Approval SMC 5138–18). The requirement for individual consent was waived because of the retrospective design and deidentified data usage. The study adhered to the Declaration of Helsinki principles.

## Results

### Population characteristics

During the Israeli–Gaza War (October 7, 2023–May 31, 2024), a total of 2,422 casualties were recorded. Among these patients, 117 (4.8%) experienced genitourinary (GU) injuries (Table 1). The mean age was 27.2 ± 9.2 y for GUs vs 30.1 ± 15.1 y for non-GUs; *p* = 0.43, with males constituting 97.4% (n = 114) (Table 1). Most of the GU patients (111, 94.9%) had associated injuries.

**Table 1:** Demographic characteristics

Variable	GU (n = 117)	Non-GU (n = 2305)	p value
Age, mean ± SD (y)	27.2 ± 9.2	30.1 ± 15.1	0.43
Range (y)	14 – 76	0–97	—
Male, %	97.4	94.3	0.15
ISS ≥ 16, %	61.5	21.6	< 0.0001

### Injury mechanisms and severity

Explosive mechanisms accounted for 59% of GU injuries, whereas gunshot wounds accounted for 37%. Severe polytrauma (ISS ≥ 16) was significantly more common among GU trauma patients (61.5%) than among non-GU patients (21.6%, p < 0.0001).

### Anatomical distribution of GU injuries

External genital injuries were the most common (60.7%), followed by kidney (27.4%) and bladder injuries (10.3%). The anatomical distribution of these injuries is detailed in Table 2.

**Table 2:** Anatomical distribution of genitourinary injuries

Anatomical Region	Frequency (n)	Percent of 117 casualties (%)
External genitalia	71	60.7
Kidney	32	27.4
Ureter	8	6.8
Bladder	12	10.3
Urethra	3	2.6
Overall Kidney/Ureter	39	33.3
Overall Bladder/Urethra	13	11.1

^‡^ Counts are patient-level. Column totals may exceed 100% because some casualties sustain multiple organ injuries within the same region

External genitalia injuries were frequent and included the following:

- Scrotum (34.2%, n = 40)
- Testes (18.0%, n = 21)
- Penis (19.7%, n = 23)
- Overall, 71 of 117 casualties (60.7%) sustained at least one external-genital organ injury. Multiple organs were affected in 13 of those patients, so the summed organ count (n = 84) exceeded the patient count.

Upper urinary tract injuries involved:

- Kidney: 27.4% (n = 32)
- Ureter: 6.8% (n = 8)
- Overall incidence of kidney and ureter injuries: 33.3% (n = 39)

Lower urinary tract injuries included the following:

- Bladder injuries (10.3%, n = 12)
- Urethral injuries (2.6%, n = 3)
- Bladder or urethral injury occurred in 13 patients (11.1%), including 2 with simultaneous bladder and urethral trauma (15 organ-level injuries).

### Combined injury patterns

Combined genitourinary injuries involving 2 or more distinct anatomical structures occurred in 17.1% (20/117) of patients. These combinations included the following:

- External genitalia combined with another GU organ: 45% (9 Patients)
  o External genitalia + Bladder: 6 patients
  o External genitalia + kidney: 2 Patients
  o External genitalia + Urethra: 1 Patients
- Bladder and urethral combined injuries: 10% (2 Patients)
- Kidney and ureter combined injuries: 5% (1 Patients)
- Multiple external genital organs involved (penis, scrotum, testes combinations): 40% (8 Patients)

### Associated pelvic fractures

Pelvic fractures were markedly more common among patients with GU injuries (18%, n = 21) than among those without GU injuries (1.8%, n = 42) (p < 0.0001) (Table 3). Of all patients with pelvic fractures (n = 63), one-third (33.3%) had a concomitant GU injury, compared with only 4.1% of patients without pelvic fractures (p < 0.0001; Cramer’s V = 0.217). Notably, bladder and/or urethral injuries were present in 12.7% of patients with pelvic fractures versus 0.2% of those without (p < 0.0001; Cramer’s V = 0.272). Within the GU injury cohort, other associated injuries included head trauma in 19.7%, thoracic injuries in 30.8%, spinal injuries in 12.8%, and lower-extremity injuries in 69.2%, with no documented upper-extremity injuries.

**Table 3:** Injury mechanisms, severity and associated pelvic fractures

Variable	GU Patients (%)	Non-GU Patients (%)	p value
Explosive	59	54.3	0.004
Gunshot	36.8	34.1	
Combined Mechanism/other	4.2	11.6	
ISS ≥ 16 (Severe/critical)	61.5	21.6	< 0.0001
ISS < 16 (Mild-Moderate)	38.5	78.4	< 0.0001
Pelvic Fractures	18	1.8	< 0.0001

### Clinical outcomes and surgical interventions

Surgical intervention was required in 84.6% of the GU patients, which was a significantly greater incidence than that in non-GU-injured individuals (53.8, p < 0.0001). Compared with non-GU-injured individuals, those with ICU admissions (44.4%), prolonged hospitalization ≥ 14 days (41.4%), rehabilitation requirements (53%), and mortality rates (6.8%) were significantly more common (all p < 0.0001; Table 4). The details of the surgical interventions are presented in (Table 5).

**Table 4:** Clinical Outcomes and Interventions

Outcome/Intervention	GU Patients (%)	Non-GU Patients (%)	p value
ICU Admission	44.4	17.8	< 0.0001
LOS ≥ 14 days	41.4	17.2	< 0.0001
Rehabilitation Required	53.0	29.2	< 0.0001
Mortality	6.8	2.0	< 0.0001
Overall Surgical Intervention	84.6	53.8	< 0.0001

**Table 5:** Detailed surgical interventions

Surgical Intervention	Frequency (n)	Percent (%)
Any urological procedure	59	50.4
Scrotal/testicular	32	27.4
Bladder/urethra	15	12.8
Penile	8	6.8
Kidney	4	3.4

Overall, 84.6% of the GU casualties underwent surgery. Specific procedures:

In the multivariable logistic regression, the presence of any GU injury remained independently associated with ICU admission (adjusted OR 2.41, 95% CI 1.14–5.10; p = 0.02) after controlling for age, injury severity score and injury mechanism (Table 6).

**Table 6:** Independent predictors of ICU admission (logistic regression)

Predictor	Adjusted OR	95% CI	p value
Any GU injury	2.41	1.14–5.10	0.02
Age (per 10 y)	1.08	0.97–1.21	0.16
ISS ≥ 16	3.56	2.01–6.33	< 0.0001
Explosive mechanism	1.27	0.79–2.06	0.32

Overall, surgical intervention, including the following:

- Any urological surgery: 50.4% (n = 59)
- Scrotal/testicular surgeries (27.4%, n = 32, including orchiectomy and testicular salvage)
- Bladder/urethral procedures (12.8%, n = 15, including primary repairs and reconstructive urethroplasty)
- Penile surgeries (6.8%, n = 8, reconstructive repairs)
- Kidney surgeries (3.4%, n = 4, primarily nephrectomies)

### Hospital course and outcomes

#### Emergency department disposition and in-hospital ICU use:

Overall ED disposition differed substantially between groups (OR vs ICU vs ward; χ^2^ test, p < 0.0001), indicating distinct destination patterns on arrival.

- Direct transfer to the operating room:
  GU: 74/117 (63.3%) vs non-GU: 573/2305 (24.9%); p<0.0001.
- Direct transfer to the ICU from the ED:
  GU: 7/117 (6.0%) vs non-GU: 146/2305 (6.3%); p=0.88. Proportions are closely similar, with no statistical difference.
- Direct admission to a standard inpatient ward:
  GU: 35/117 (29.9%) vs non-GU: 1572/2305 (68.2%); p<0.0001. Ward admissions comprised the minority of ED dispositions in GU and the majority in non-GU.
- ED deaths: GU: 1/117 (0.85%) vs non-GU: 13/2305 (0.56%); events were rare in both cohorts.

#### Any ICU use during hospitalization (beyond the ED)

GU: 44.4% vs non-GU: 17.8%; two-proportion test, p<0.0001. This measure reflects critical- care utilization at any point during the admission, not solely ED disposition.

#### Length of hospital stay (LOS)

- Prolonged hospitalization (≥ 14 days): GUs 41.4% vs. non-GUs 17.2% (p < 0.0001).
- Short stays (≤ 3 days): GUs 25.9% vs. non-GUs 52.8%.

#### Discharge outcomes and mortality

- Direct home discharge was lower among GUs (38.5%) than among non-GUs (67.7%) (p < 0.0001).
- Rehabilitation was significantly more common in GU patients (53% vs. non-GU 29.2%, p < 0.0001).
- Mortality was significantly greater: GU, 6.8%; non-GU, 2.0%; p < 0.0001).

## Discussion

This study provides a detailed characterization of war-related genitourinary trauma sustained during the Israeli–Gaza War, with emphasis on injury patterns, operative management, resource utilization, and short-term clinical outcomes. Although GU trauma affected fewer than 5% of casualties, these injuries were disproportionately severe, with major implications for patient morbidity, long-term functional outcomes, and healthcare resource allocation.

### Clinical implications and injury patterns

The predominance of external genitalia injuries (60.7% overall genital involvement) is consistent with international conflict data, underscoring persistent vulnerability due to insufficient protective gear. Similar findings were reported from the ISAF mission in Afghanistan, where external genital trauma was also the most frequent urological injury [12].

In the context of our cohort, this vulnerability is further amplified by the absence of dedicated pelvic and gonadal protective equipment in routine issue within the Israel Defense Forces, a circumstance that may directly contribute to the high incidence of these specific injuries. This highlights the urgent requirement for enhanced pelvic protection specifically designed to shield the scrotum and testes. Similar observations were reported in Operation Iraqi Freedom and Operation Enduring Freedom (OIF/OEF), where a high proportion of external genitalia injuries occurred despite the use of modern body armor [2, 4, 10]. Our findings therefore support calls for targeted improvements in protective equipment to address this ongoing gap.

Upper urinary tract injuries, particularly renal-ureteric trauma (33.3%), emphasize the importance of rapid urological imaging and coordinated surgical management to maximize renal preservation and reduce long-term complications.

Lower urinary tract injuries, particularly bladder-urethral injuries (11.1%), present substantial reconstructive challenges, reinforcing the need for prompt urological referral and reconstructive expertise in accordance with the 2025 EAU Guidelines on Urological Trauma [14].

Combined genitourinary injuries were observed in 17.1% of our cohort—including simultaneous external genital injuries with bladder (n = 6), kidney (n = 2), or urethral injuries (n = 1), as well as bladder-urethra (n = 2) and kidney-ureter (n = 1) combinations—reflecting significant anatomical complexity and management challenges. This pattern mirrors previous reports from OIF/OEF, where high-energy explosive mechanisms frequently produced complex multiorgan rather than isolated injuries [2, 4]. Such injury constellations underscore the necessity of coordinated multidisciplinary care involving urologists, trauma surgeons, and orthopedic specialists [2, 4, 10], and highlight the need for trauma systems with robust protocols to manage these resource-intensive, rehabilitation-heavy cases.

### Pelvic fractures and associated injuries

The strong association between pelvic fractures and concomitant GU injury in our study (33% vs. 4% without pelvic fracture) mirrors findings from both civilian and military trauma series, reflecting the anatomical proximity and shared high-energy mechanisms of injury. The rate of bladder and/or urethral trauma in pelvic fracture patients (12.7%) was markedly higher than the 0.2% without pelvic fracture, consistent with established evidence identifying pelvic fractures—especially from blast mechanisms—as a key risk factor for lower urinary tract disruption.

The high prevalence of other associated injuries in the GU cohort—lower extremity injuries (~ 70%), thoracic injuries (31%), and head injuries (20%)—confirms that modern wartime GU trauma typically occurs as part of complex multisystem injury patterns, reinforcing the need for coordinated multidisciplinary care.

### Surgical management and multidisciplinary approach

Our cohort had a high surgical intervention rate (84.6%), consistent with the complex injury mix seen in modern conflicts and comparable to the 79–86% reported by Hudak and Serkin in U.S. and NATO forces [2, 4]. Of these, only 46.2% were urinary tract–specific procedures, underscoring the frequent need for shared operative management with orthopedic, vascular, and general surgery teams.

The operative versus conservative treatment distribution closely followed the 2025 EAU Guidelines on Urological Trauma [14]:

- *Kidney*: Renal injury was the most common upper-tract lesion (33.3%), yet nephrectomy was performed in only 3.4% (n = 4) of patients, aligning with the guideline’s preference for nonoperative management (NOM) in stable cases, and selective use of angioembolization or surgery for ongoing hemorrhage.
- *Ureter:* Rare (6.8%); all complete transections underwent immediate segmental repair, in line with the EAU recommendation for early reconstruction over delayed diversion.
- *Bladder:* Intraperitoneal ruptures were repaired operatively; extraperitoneal tears were managed with catheter drainage alone, matching guideline recommendations and supporting the organ preservation approach applied in 54/117 patients.
- *Urethra:* All pelvic-ring disruption cases had same-day retrograde urethrography; complete posterior disruptions were managed with suprapubic diversion and delayed urethroplasty, reflecting the 2025 shift away from immediate primary repair.
- *External genitalia:* All suspected testicular ruptures underwent urgent exploration, and penile fractures were repaired promptly, consistent with EAU algorithms linking early intervention to optimal outcomes.

## Hospital Course, Resource Utilization, and Severity Profile

Consistent with the injury pattern described above, the ED destination profile also diverged meaningfully between groups. GU casualties were routed far more often directly to the operating room (63.3% vs. 24.9%; p < 0.0001) and far less often to routine wards (29.9% vs. 68.2%; p < 0.0001), whereas direct ED-to-ICU admission was similar (6.0% vs. 6.3%; p = 0.88). Over the course of the admission, however, any ICU use was substantially higher in GU patients (44.4% vs. 17.8%; p < 0.0001), and GU injury remained independently associated with ICU utilization (adjusted OR 2.41). Taken together, these data delineate a severity signature characterized by high procedural acuity at presentation and greater critical-care exposure across the stay—without evidence of increased immediate ICU triage at the door. This profile aligns with the ISAF experience reported by Schoch et al. [12] and echoes contemporary DoDTR [13] analyses that emphasize downstream resource burdens in survivors with genital and associated injuries.

### Mortality and comparative analysis

The higher mortality rate among GU trauma patients (6.8%) compared with non-GU injured individuals reflects the severity and complexity of the polytrauma frequently associated with these injuries. Although lower than rates reported in some historical conflicts, a finding attributable to significant advances in combat casualty care over the past two decades, it remains higher than in non-GU trauma patients.

The 4.8% incidence of GU injuries in our series aligns with the 1–5% range reported in prior conflicts, including OIF and OEF]1,2,11[. For example, Belmont et al. (2010) documented the epidemiology of combat wounds in OIF/OEF, including GU injuries]11[.

The asymmetric warfare context of our study—with frequent explosive devices and rocket fire in civilian areas—likely contributed to the high proportion of external genitalia injuries (60.7%), a finding consistent with prior work highlighting pelvic vulnerability despite modern body armor]4[.

The predominance of explosive mechanisms (59%) compared with gunshot wounds (37%) underscores the destructive potential of these injuries and their link to severe polytrauma (61.5% with ISS ≥ 16), as also noted by Hudak et al. (2013)]2[. A recent systematic review reinforced the evolving approach to bladder trauma in war injuries]7[.

Our high surgical intervention rate (84.6%) reflects the complexity of these injuries, which frequently require advanced reconstructive procedures. This parallels the shift in battlefield urology toward organ-sparing and complex reconstruction to preserve function, as reported by Elliott et al. (2017) [15].

The elevated ICU admission rate (44.4%), prolonged hospitalization (41.4%), and substantial rehabilitation needs (53%) highlight the heavy healthcare resource demands associated with wartime GU trauma, consistent with prior conflict data. Eastridge et al. (2012) stressed the need for robust trauma systems and rehabilitation pathways to meet these demands]8[.

While mortality remains a concern, these findings underscore the need for ongoing advances in protective equipment, triage algorithms, and multidisciplinary management to optimize outcomes in war-related GU trauma.

## Strengths and limitations

This study has several strengths, including the use of a large national trauma registry, which provides a comprehensive overview of GU injuries during the Israeli–Gaza War. The data are systematically collected and audited, enhancing their reliability. This study also provides valuable information on the injury patterns, management strategies, and clinical outcomes of these injuries, which can inform clinical practice and future research.

However, the study also has several limitations. As a retrospective study, it is subject to the inherent limitations of this design, including potential underreporting or incomplete data, particularly in the context of a mass-casualty event. The reliance on registry data may introduce selection bias, as the registry may capture a greater proportion of severely injured patients since it includes only hospitalized patients. Variability in diagnostic imaging and incomplete documentation under battlefield conditions may contribute to data gaps, necessitating cautious interpretation of milder injury prevalence and management strategies. In addition, the registry lacks prehospital/damage-control variables and protocol-adherence fields; therefore, we did not undertake formal comparisons with combat-specific Clinical Practice Guidelines (e.g., JTS) and deliberately confined interpretation to in-hospital findings. Future studies should aim to validate these findings with data from other sources and employ prospective data collection methods.

The registry did not capture sufficient detail to differentiate between specific types of explosive devices (e.g., IED vs. other explosives), which limits our ability to analyze injury patterns in relation to distinct blast mechanisms.

## Future directions and recommendations

Prospective, standardized data collection in multicenter studies remains essential. Further research should focus on the following:

- Identifying specific risk factors for GU injuries in different combat scenarios.
- Evaluating the effectiveness of different surgical techniques and treatment protocols.
- Assessing the long-term functional and psychosocial outcomes of patients with GU trauma.
- The efficacy of new protective gear and strategies to prevent these injuries should be developed and evaluated.

Addressing these research gaps will require collaborative efforts and international cooperation to improve the prevention and management of war-related GU trauma and minimize its devastating impact on affected individuals.

## Conclusions

War-related GU trauma, though comprising a relatively small percentage of overall casualties in the Israeli-Gaza War, represents a category of particularly severe injuries. These injuries are associated with a high likelihood of requiring extensive surgical management, lead to prolonged hospitalizations, and necessitate substantial post-discharge rehabilitation. The findings underscore the critical need for ongoing development and implementation of enhanced protective equipment specifically for the pelvic region, alongside the continued refinement of multidisciplinary treatment strategies to optimize outcomes for casualties in current and future conflicts.

## Data Availability

The datasets generated and/or analysed during the current study are not publicly available due to patient privacy considerations and institutional data sharing policies of the Israeli National Trauma Registry. However, anonymized data may be available from the corresponding author on reasonable request and with the explicit permission of the Israeli National Trauma Registry and the Chaim Sheba Medical Center Institutional Review Board.

## Abbreviations

AIS: Abbreviated Injury Scale
EAU: European Association of Urology
GU: Genitourinary
ICU: Intensive Care Unit
IED: Improvised Explosive Device
INTR: Israeli National Trauma Registry
DoDTR: Department of Defense Trauma Registry
IQR: Inter-quartile Range
ISS: Injury Severity Score
ITG: Israel Trauma Group
LOS: Length of Stay
NOM: Non-operative Management
OEF: Operation Enduring Freedom
OIF: Operation Iraqi Freedom
OR: Odds Ratio
SD: Standard Deviation
